# Gonadotropins in the Russian Sturgeon: Their Role in Steroid Secretion and the Effect of Hormonal Treatment on Their Secretion

**DOI:** 10.1371/journal.pone.0162344

**Published:** 2016-09-13

**Authors:** Svetlana Yom-Din, Lian Hollander-Cohen, Joseph Aizen, Benjamin Boehm, Michal Shpilman, Matan Golan, Avshalom Hurvitz, Gad Degani, Berta Levavi-Sivan

**Affiliations:** 1 Department of Animal Sciences, The Robert H. Smith Faculty of Agriculture, Food and Environment, The Hebrew University of Jerusalem, Rehovot, 76100, Israel; 2 MIGAL Galilee Technology Center, PO Box 831, Kiryat Shmona, 10200, Israel; Tel Aviv University, ISRAEL

## Abstract

In the reproduction process of male and female fish, pituitary derived gonadotropins (GTHs) play a key role. To be able to specifically investigate certain functions of Luteinizing (LH) and Follicle stimulating hormone (FSH) in Russian sturgeon (*Acipenser gueldenstaedtii*; st), we produced recombinant variants of the hormones using the yeast *Pichia pastoris* as a protein production system. We accomplished to create in vitro biologically active heterodimeric glycoproteins consisting of two associated α- and β-subunits in sufficient quantities. Three dimensional modelling of both GTHs was conducted in order to study the differences between the two GTHs. Antibodies were produced against the unique β-subunit of each of the GTHs, in order to be used for immunohistochemical analysis and to develop an ELISA for blood and pituitary hormone quantification. This detection technique revealed the specific localization of the LH and FSH cells in the sturgeon pituitary and pointed out that both cell types are present in substantially higher numbers in mature males and females, compared to immature fish. With the newly attained option to prevent cross-contamination when investigating on the effects of GTH administration, we compared the steroidogeneic response (estradiol and 11-Keto testosterone (11-KT) in female and males, respectively) of recombinant stLH, stFSH, and carp pituitary extract in male and female sturgeon gonads at different developmental stages. Finally, we injected commercially available gonadotropin releasing hormones analog (GnRH) to mature females, and found a moderate effect on the development of ovarian follicles. Application of only testosterone (T) resulted in a significant increase in circulating levels of 11-KT whereas the combination of GnRH + T did not affect steroid levels at all. The response pattern for estradiol demonstrated a similar situation. FSH levels showed significant increases when GnRH + T was administered, while no changes were present in LH levels.

## Introduction

Sturgeons are one of the most primitive vertebrates inhabiting cold freshwater rivers and lakes. With over 200 million years of history, they represent a key position in evolution. As their roe is a source of caviar, sturgeon have high economic value, which has become the primary factor for their exceedingly endangered status due to overfishing [1].

The accepted model for the role of gonadotropins (GTH) in fish comprise that follicle-stimulating hormone (FSH) is involved in the regulation of early gametogenesis while luteinizing hormone (LH) stimulates processes leading to final oocyte maturation and ovulation in females, and spermiation in males (reviewed by [2]. The duality of gonadotropic activity in fish was determined in the 1980s [3]. As in mammals, these gonadotropic hormones were purified from the pituitary and shown to be heterodimers ranging from 30 to 50 kDa. These heterodimeric glycoproteins were found to be composed of two non-covalently associated subunits [2]. The production of specific antibodies indicated that glycoprotein hormone α subunit (GPα) was common in both hormones, while the β subunit was found to differ for each hormone (FSHβ or LHβ) [2]. In the last decade, the number of isolated and characterized cDNAs encoding fish gonadotropin (GTH) subunits has greatly increased. This allowed the production of species-specific recombinant fish GTHs in heterologous systems enabling their continuous availability. Furthermore, cross-contamination with other related glycoproteins is prevented [2].

Traditionally, GTH levels in fish have been determined by radioimmunoassay (RIA) or enzyme-linked immunosorbent assay (ELISA) based on native GTHs isolated from fish pituitaries and their specific antibodies. However, the purification of native GTHs is a resource demanding process. The requirement of large numbers of pituitaries, costs and labor makes it a second choice approach [2].

Homologous immunoassays for FSH have been developed for several fish species, including rainbow trout (*Oncorhynchus mykiss*) [4–6], Nile tilapia (*Oreochromis niloticus*) [7], killifish (*Fundulus heteroclitus)* [8], European bass (*Dicentrarchus labrax*) [9] and Senegalese sole (*Solea senegalensis*) [10]. For a wide range of fish species, quantitative analysis methods for LH are available, a fact which enabled only an incomplete endocrine description, especially regarding the role of FSH in fish. However, molecular methods enable the isolation and characterization of cDNAs encoding GTH subunits in different fish species that were further used for the preparation of species-specific recombinant GTHs. Recombinant GTHs can serve as a useful substitute to native hormones, since they can be produced in larger quantities that will be accessible and will not cross-react with other pituitary hormones [2].

Sturgeons are late-maturing fish, with the females reaching puberty under aquaculture conditions at the age of 6 to 10 years, depending on the species and culture conditions. In the first year of maturity, only 5 to 10% of the females will be ready for caviar production; the remaining females will take 1, 2 or even 3 more years to mature. This asynchronous first maturation makes it difficult for caviar producers to manage livestock and plan production and sales [11,12]. The initiation of the reproductive act is primarily correlated with increased secretion of hypothalamic gonadotropin-releasing hormone (GnRH), whereas the onset of gonadal development is accompanied by increased steroid production

Studying the pituitary glycoprotein hormones in basal and later evolved teleosts has great potential to provide valuable insights of the molecular mechanisms of these crucial hormones. We investigated the prolonged effect of GnRH combined with the effect of testosterone (T) on the synchronization of puberty in immature female sturgeon. The objectives of this study were to: i) produce glycosylated, biologically active sturgeon GTHs (stGTHs) using an expression system capable of producing large quantities of bioactive recombinant proteins; ii) create specific antibodies against the stGTHs and utilize them to establish and validate a homologous competitive ELISA; iii) study the effects of the novel recombinant stGTHs on steroid release; iv) study the effects of combined GnRH + T treatment on the release of stGTHs and steroids in mature females.

## Materials and Methods

### Fish

Russian sturgeons (*Acipenser gueldenstaedtii*) originated in the Caspian Sea were brought from Russia and reared from eggs at “Dan Fish Farms” (Upper Galilee, Israel; 31°30′N, 34°45′E) under aquaculture conditions. Fish were maintained in 250- to 500-m^3^ concrete tanks (water temperature (14–24°C) and 12h photoperiod) and were fed twice a day with 4-mm pelleted feed (trout feed; Zemach Feed Mills, Zemach, Israel, containing 42% protein and 12% fat) at 0.5–1% (by weight) of their biomass, depending on the season.

To analyze the recombinant GTHs' biological activity, 10-year-old female sturgeon were sampled in August, when water temperature was 22 ± 0.5°C. Each fish was anesthetized in a clove oil bath (0.25 mg/L), weight and length were recorded, and blood was taken from the caudal vasculature using heparinized syringes. After centrifugation (3000 rpm for 30 minutes at 4°C), the plasma was stored at -20°C until processing.

All experiments were conducted in accordance with the Animal Care and Use Guidelines and were approved by the National Research Council for Care and Use of under permit number IL-15-12-391. The study was carried out at the private commercial sturgeon farm "Caviar Galilee", owned by Kibbutz Dan, and authorized by the CEO of Caviar Galilee. The field portions of the study were conducted only on the farm premises, therefore, no additional permissions were required. The Russian sturgeon is protected by the international convention CITES. However, Caviar Galilee operates under full control of the CITES authority in Israel—The Authority for the protection of nature and national parks.

### Experimental design that study the effect of long-term T and/or GnRHa treatments on the pituitary–ovary axis of prepubertal female sturgeon

To determine the combined effect of GnRH and T on GTH secretion, 6-year-old female fish were divided into three groups, 10–13 fish per group, with a mean body weight (BW) of 11.08 kg. The control group was injected with 0.2 mL/kg saline. The fish from the second group were administered with salmon GnRH ([D-Arg^6^, Pro^9^—NEt]-salmon GnRH (sGnRHa); Bachem Inc., Torrance, CA] in slow-release implants (Evac) to a final dose of 10 μg/kg, according to [13]. Implantation was executed into the dorsal musculature using a 2.5-mm AVID (American Veterinary Identification Devices) gun implanter; in addition to GnRH implants, the fish received T as food supplements (60 mg/kg food; 0.4% BW food per day). The same concentration of T in the food was administered to the third group. Fish were bled four times: at the start of the experiment (time 0), and monthly thereafter. At time 0 and 10 months later, all fish ovaries were internally observed by endoscopy according to (30). The ovarian stage of the biopsied ovaries was determined according to Table 1.

**Table 1:** Characteristics of ovarian stages.

Stage	Diameter (μm)	Follicle color
Pre-vitellogenic	< 600	Semi-transparent
White follicles	600–1000	White
Yellow follicles	1000–1600	Yellow
Gray follicles	1600–2600	Gray
Black follicles	2600–3400	Black

### Recombinant DNA technology, construction of the expression plasmid and protein expression

**Fig 1:**
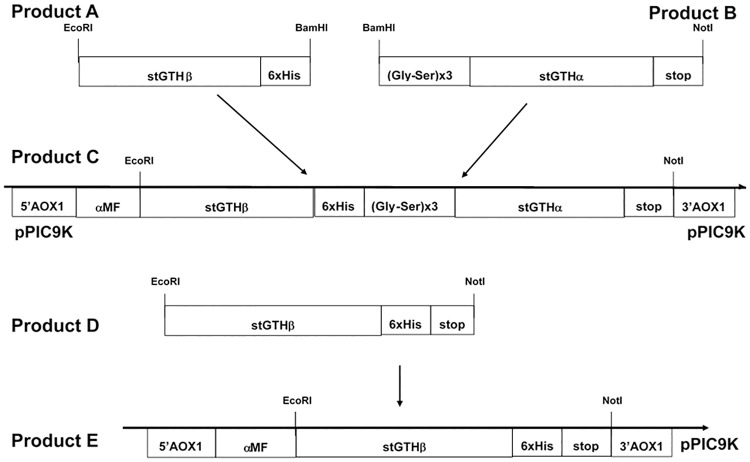
Construction of the expression vectors for the Russian sturgeon gonadotropins (GTH). Russian sturgeon (st) FSHβ, LHβ, cgα, FSHβα or LHβα were expressed in the expression vector pPIC9K that contains sequences required for selection in each host. It has the 5’ promoter and 3’ transcription termination sequences of the alcohol (methanol) oxidase gene (AOX1) flanking the cloning site into which the sturgeon gonadotropin subunits were introduced. The vector has a yeast mating factor signal peptide downstream of the AOX1 promoter to which the recombinant proteins were fused.

Amplification of the α- and β-subunit sequences of stLH and stFSH, subcloning of fused genes into a yeast (*Pichia pastoris*) expression vector and the expression of recombinant stGTHs were performed following the same procedure used to express tilapia LHβα [14] and tilapia FSHβα [15]. Sequences of the known Russian sturgeon α-glycoprotein subunit gene (*cga*), *fshb* and *lhb* (GenBank Accession nos. AY519658, AY519657, AY333426, respectively; [16] were codon-optimized according to the codon usage of the expression system *P*. *pastoris*. The optimized synthetic genes were synthesized by Thermo Fisher Scientific GENEART GmbH (Regensburg, Germany) and fusion genes were formed and subcloned into the *Pichia* expression vector. The synthetic genes encoding the mature region were joined to form a fusion gene that encodes a "tethered" polypeptide in which one of the β chains forms the N-terminal domain and the α chain forms the C-terminal domain. A "linker" sequence of six amino acids (three Gly–Ser pairs) was placed between the β and α chains to assist in chimerization of the subunits, and a six-His (His^6^) tail was placed at the end of the β subunit to enable purification of the recombinant protein (Fig 1). The gene encoding stLHβ or stFSHβ (containing a His^6^-tag at the C terminus of the β subunit; Product D in Fig 1) was cloned into the pPIC9K vector (Invitrogen, Carlsbad, CA) (Product E in Fig 1), while stFSHβα or stLHβα [containing a His^6^-tag at the C terminus of the β subunit (Product A in Fig 1) and a linker at the N terminus of the α subunit (Product B in Fig 1)] was cloned into the *Eco*RI*-Not*I sites of the pPIC9K vector, giving rise to Product C (Fig 1). The pPIC9K plasmid contained the α-factor secretion signal that directs the recombinant protein into the secretory pathway. The constructs were digested with *Sal*I and used to transform *P*. *pastoris* strain GS115 by electroporation (Product C and E in Fig 1, for stGTHβα and stGTHβ, respectively). This resulted in insertion of the construct at the *HIS4* locus of *P*. *pastoris*, generating a His^+^ Mut^+^ phenotype. Transformants were selected for the His^+^ phenotype on 2% agar containing dextrose–biotin regeneration medium (1 M sorbitol, 2% dextrose, 1.34% yeast nitrogen base, 4 x 10^−5^% biotin, and 0.005% L-Glu, L-Met, L-Lys, L-Leu, and L-Ile), and then further selected for high copy number by their growth rate on 2% agar containing 1% yeast extract, 2% peptone, 2% dextrose medium, and the antibiotic G418 at various concentrations (0.5–2 mg/mL; Invitrogen). The protein was expressed in a shaker flask and harvested 72 h after induction by methanol. Recombinant proteins were purified using nickel-nitrilotriacetic acid agarose (Ni-NTA; Qiagen, Alameda, CA) according to [14]. As a negative control, *P*. *pastoris* was transformed with an expression vector that did not contain the recombinant protein and fractions were prepared in the same manner.

### Sturgeon gonadotropin antiserum production

The antiserum against recombinant stFSHβ and stLHβ was raised in rabbit by intradermal injection (according to (13)) with stFSHβ or stLHβ emulsified in an equal volume of complete Freund’s adjuvant. Injections were performed four times at 3-week intervals (50 μg in each injection). The rabbits were bled 2 weeks after the final injection, and the serum was aliquoted and lyophilized.

### Gel electrophoresis and western-blot analysis

Native non-reduced samples of sturgeon pituitary extract (StPE) and recombinant proteins were electrophoresed on 15% polyacrylamide running gels with a 5% stacking gel. Gels were blotted onto nitrocellulose membranes (Schleicher and Schuell, Dassell, Germany), and blocked with 5% low-fat milk; StPE, stFSHβ, stFSHβα, stLHβ and stLHβα were visualized with the antisera against recombinant stFSHβ or stLHβ (Section 2.3) and with anti-His. The membranes were incubated in PBS plus 1% non-fat milk with the antisera (1:2,000) for 1 h at room temperature (RT), and then with goat anti-rabbit horseradish peroxidase conjugate (GAR–HRP; Jackson, ImmunoResearch Laboratories, Inc. West Grove, PA; 1:10,000) for 1 h at RT. After washing, all membranes were treated with enhanced chemiluminescence reagent (ECL; Biological Industries, Beit Ha’emek, Israel) to reveal immunoreactive bands.

### Structural modeling of stFSH and stLH

The amino acid sequences of the sturgeon glycoprotein subunits GPα, LHβ and FSHβ used for the modeling were extracted from NCBI with GenBank Accession nos. AAS92716, AAP97490 and AAS92715, respectively [16]. Models of stLH and stFSH were prepared using the I-TASSER server (a bioinformatics method for predicting 3D structural models of protein molecules: http://zhanglab.ccmb.med.umich.edu/I-TASSER/) [17,18]. For the ligand models, the default parameters were used, with the human (h) FSH structure 1FL7 as the template. Visualization and superpositioning of the models were performed using PyMOL v1.7.6 (Schrödinger).

### *In-vitro* bioassay of sturgeon gonads

Testes from 2-year-old (prepubertal; n = 9) and 4-year-old (mature; n = 7) male sturgeons (875 ± 137.1 g and 7420 ± 1825 g, mean ± SEM, BW; 9.32 ± 3.75 g and 57 ± 13.84 g gonadal weight; GSI (gonadosomatic index; gonad mass as a percentage of the total BW) 0.15% ± 0.03% and 0.73% ± 0.03%, respectively) were divided into uniformly sized fragments (of about 40 mg each). Since female sturgeon has a group-synchronous ovarian development, ovaries from 10-year-old sturgeons (19,200 ± 5,135 g mean ± SEM, BW; 3,312 g ±805 g mean ± SEM gonadal weight; GSI = 17.25% ± 6.4%; n = 12), were manually separated for isolation of specific stages according to their size and color (Table 1). Gonads were collected immediately upon sacrificing the fish and incubated according to previously described procedures [15]. Briefly, gonads were placed in a 90-mm culture dish and washed several times with basal medium Eagle (BME) containing NaHCO_3_ (4 mM), penicillin (100 IU/mL), streptomycin (0.1 mg/mL) and nystatin (1.25 IU/mL) (Biological Industries; Beit Haemek, Israel) and 0.05% bovine serum albumin (BSA; SIGMA, Rehovot, Israel) and buffered to pH 7.4 with 2.1 mM HEPES. The gonads were pre-incubated by frequent rinsing for 2 h in a 48-well culture plate at 28°C (1 mL/well). Then, the medium was replaced with the tested recombinant GTH diluted in the same medium to form graded concentrations ranging from 0.5 ng/mL to 1000 ng/mL for an incubation time of 16 h. These were performed in triplicate wells per treatment and were repeated twice to confirm the results. In order to be able to compare the relative activities of the recombinant GTHs, results are expressed as percentage over the basal levels.

### Establishment of specific ELISAs for the determination of stFSH and stLH

Competitive ELISAs for determinations of stLH and stFSH (generally according to [7]) were developed using primary antibodies against specific β subunits (Section 2.3), recombinant β subunit (Section 2.2) to coat the ELISA microplates, and recombinant stLHβα or stFSHβα (Section 2.2) for the standard curve. Briefly, ELISA microtiter plates (Nunc-Immuno Plates, Nunc, Denmark) were coated with 100 μL/well of 5 ng/mL (0.5 ng/well) stFSHβ or 20 ng/mL (2 ng/well) stLHβ. The next day, plates were washed with 200 μL/well PBST buffer [10 mM Na_2_HPO_4_, 2 mM KH_2_PO_4_ (pH 7.4), 140 mM NaCl, 3 mM KCl, and 0.05% Tween 20]. To reduce the background, plates were blocked for 1 h with 200 μL/well PBST buffer containing 1% BSA. Unknown samples and standards were first pre-incubated overnight at RT with the primary antibodies (final dilution 1:10,000 for stFSH and 1:5,000 for stLH in 0.1% BSA in PBST) in 96-well microtest plates (Sarstedt, Nümbrecht, Germany) without shaking. After pre-incubation, each sample was dispensed into the wells of the coated microtiter plates and incubated for 3h at RT. Following the incubation, the plates were washed with PBST. The formed antigen–antibody complexes were detected by addition of 100 μL/well of GAR–HRP diluted 1:5000 in PBST–0.1% BSA buffer for 2 h. The plates were washed again with PBST. The presence of enzyme complexes was visualized by addition of 100 μL/well of 3,30,5,50-tetramethylbenzidine (TMB) peroxidase substrate (KPL, Gaithersburg, MD) diluted 1:4. The reaction was carried out in complete darkness at RT and was stopped after 15 min with TMB stop solution (100 μL/well). Absorbance was read at 450 nm, using a Spectra II ELISA reader (SLT, Salzburg, Austria).

Intra-assay coefficient of variation (CV) was determined by assaying eight replicates of one of the standard concentrations (1.25 ng/mL) on the same assay plate. Inter-assay CV was determined by assaying the same sample seven times in different plates.

The ELISA was validated for stLH and stFSH determinations in plasma samples and pituitary extract of sturgeon. Displacement curves for plasma and pituitary samples were obtained by serial dilutions of the sample in the ELISA buffer and comparison with the corresponding standard curve.

### ELISAs for steroids

Estradiol (E2) and 11-ketotestosterone (11-KT) levels were determined by ELISA according to [19], using acetylcholinesterase as a label. The anti-11-KT was donated by Dr. D.E. Kime (Sheffield, UK) and is described in [20]. The antiserum against 11-KT showed 6.8% cross-reaction with T (19). The anti-E2 was described in [21]. All samples were analyzed in duplicate, and a separate standard curve was run for each ELISA plate. The lower limits of detection were 0.93 and 0.50 pg/mL for 11-KT and E2, respectively. The intra- and inter-assay CVs were less than 7 and 11%, respectively. Steroid levels in the sturgeon plasma, determined by ELISA, were validated by verifying that serial dilutions were parallel to the standard curve.

### Immunohistochemistry of sturgeon pituitaries

Immunofluorescent staining for stFSH and stLH was generally performed according to [22,23]. Briefly, tissue samples were fixed in 4% paraformaldehyde, put into cryoprotection agent (30% sucrose) and frozen in OCT (Optimal Cutting Temperature) embedding compound. Sections (15 μm) were obtained on a cryostat and collected onto superfrost slides. The sections were blocked with 5% normal goat serum for 1 h to reduce non-specific reactions. They were then incubated with rabbit anti-stFSHβ (diluted 1:250) or with rabbit anti-stLHβ (diluted 1:500) for 16 h at 4°C. Antibodies were diluted in PBS with 1% BSA and 0.3% Triton X-100. The slides were rinsed three times with PBS for 5 min and were incubated for 2 h at room temperature with goat anti-rabbit antibodies conjugated to Alexa488. Sections were then counterstained with DAPI nuclear staining. After washing, slides were mounted with anti-fade solution (2% propyl gallate, 75% glycerol, in PBS) and imaged with an epifluorescence microscope.

### Statistical analysis

Data are presented as means ± SEM. The significance of differences between group means of hormone levels was determined by one-way analysis of variance (ANOVA) followed by Newman–Keuls test using Graph-Pad Prism 4.02 software (GraphPad, San Diego, CA). To test for parallelism between various regressions lines, we used the analysis of covariance at: http://home.ubalt.edu/ntsbarsh/Business-stat/otherapplets/ANOCOV.htm. For data calculations in the ELISA, sigmoid curves were linearized using the logit transformation: logit(B/Bo) = log [r/(1−r)], where r = B/Bo, B represents the binding percentage at each point, and Bo is the maximum binding.

## Results

### Recombinant stFSHβα, stFSHβ, stLHβα, and stLHβ

In order to develop specific homologous ELISAs for the determination of stFSH and stLH, we expressed both β subunits and both heterodimers in the yeast *P*. *pastoris*. Western-blot analysis was performed on sturgeon pituitary extract and the supernatant derived from yeast transformed with an expression vector containing stFSHβα, stFSHβ, stLHβα, or stLHβ cDNAs. These proteins were immunoreacted with either anti-His or specific antibodies developed against the β subunit of each of the GTHs (see Section 2.3).

**Fig 2:**
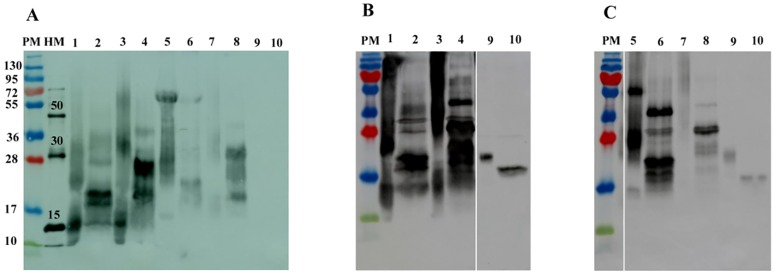
Characterization of Pichia-expressed recombinant stLHβ, stLHβα, stFSHβ and stFSHβα by Western-blot analysis and deglycosilation. Supernatants of transformed Pichia cultures were separated by 15% semi-native PAGE and immunoreacted with antibodies raised against anti-His (A) recombinant stLHβ (B) or recombinant stFSHβ (C). For deglycosylation analysis, denatured and reduced proteins were incubated with (lanes 2, 4, 6, 8 and 10) or without (lanes 1, 3, 5, 7 and 9) N-glycosidase F. stLHβ: lanes 1,2; stLHβα: lanes 3,4; stFSHβ: lanes 5,6; stFSHβα: lanes 7,8; sturgeon pituitary extract (diluted 1:100): lanes 9,10; PM-protein marker; HM-His-tag protein marker.

To facilitate purification of the recombinant protein from the culture medium, a His^6^-tag was introduced at the C-terminal end of the β subunit in each construct. The constructs were cloned into pPIC9K to obtain the constructs pPIC9KstLHβα, pPIC9KstFSHβα, pPIC9KstLHβ and pPIC9KstFSHβ (Products C and E, respectively, Fig 1). The yeast transformants were selected, screened and purified as previously described [14,15]. The yield of the recombinant proteins was 540 μg/liter and 1570 μg/liter for stFSHβα and stLHβα, respectively. Under reducing conditions, the immunoreactive stLHβ, stLHβα, stFSHβ and stFSHβα were revealed with both anti-His (Fig 2A) and the respective antisera (Fig 2B for LH and Fig 2C for FSH). The stLHβ/stFSHβ showed bands of ~22 kDa and the stLHβα/stFSHβα showed bands of ~32 kDa (Fig 2). When the homogenate of vitellogenic female sturgeon pituitaries was subjected to SDS-PAGE under reducing conditions, a protein of approximately 20 kDa reacted specifically with anti-stLHβ and anti-stFSHβ and, not surprisingly, no reaction was revealed with the anti-His (Fig 2). Transformation with the vector alone served as a negative control (data not shown).

After deglycosylation with PNGase F of the asparagine-linked glycans, anti-His, anti-stLHβ and anti-stFSHβ were reacted with the ~20-kDa proteins of stLHβ/stFSHβ and with the ~28-kDa proteins of stLHβα/stFSHβα (Fig 2). All four proteins showed a clear decrease in molecular mass by deglycosylation of the *N*-glycosylation type. Deglycosylation of the pituitary proteins yielded a band at around 18 kDa for the heterodimers. In addition to the main bands, the stLHβ/stFSHβ supernatant and the deglycosylated stLHβα/stFSHβα supernatant contained labeled bands of 15 kDa and 17 kDa, respectively (Fig 2). The difference in estimated molecular masses probably resulted from differences in the degree of glycosylation.

### The structures of recombinant stFSH and stLH

The stFSH, stLH, and subunit α and β conformations generated by I-TASSER were superposed on the existing structures of representative hGTHs extracted by the PDB database. The hGTH structures used were: hCGα (human Chorionic Gonadotropin) PDB entries 1E9J and 1DZ7 [24,25], hCG PDB entries 1QFW and 1HCN [26], hFSH PDB 1FL7 [27] and hFSH bound to hFSH receptor–extracellular domain (hFSHR–ECD) PDB 1XWD [28]. PyMOL superposition by sequence alignment was used for comparison of the available structures.

**Fig 3:**
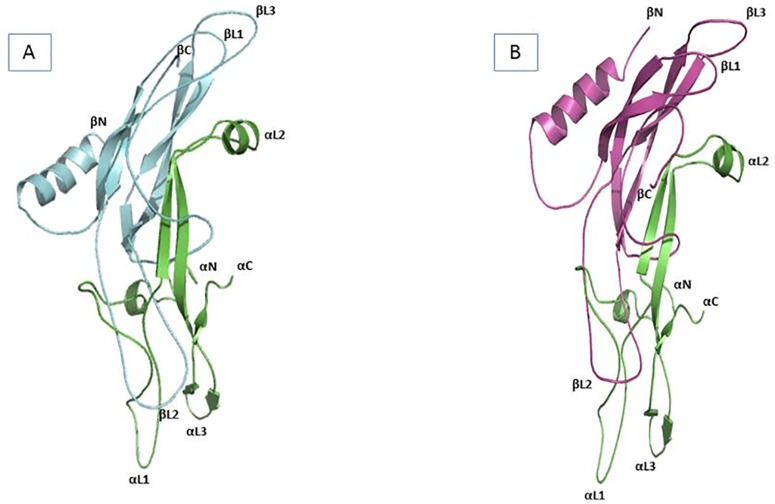
Russian sturgeon gonadotropin models. A. stFSH model. B. stLH model. Red colored: Subunit α, blue colored: Subunit β, αC: subunit α C-termini, αN: subunit α N- termini, βC: Subunit β C- termini, βN: subunit β N- termini.

Superposition of stFSH and stLH on the existing hGTH structures showed high similarity among stGTH β subunits, hCG and hFSH, with a backbone RMSD (root mean square deviation) of 0.78 to 2.46 Å. Specifically, hCG structure 1QFW gave the lowest RMSD values (0.78 Å and 0.79 Å for stFSHβ and stLHβ, respectively), while hFSH structure 1FL7 gave the lowest RMSD values for stFSHβ (1.54 Å) and stLHβ (2.46Å). The GPα gave an RMSD value 0.78 Å when superposed on hFSH structure 1WXD, and 0.82 Å when superposed on hCG structure 1QFW (Fig 3).

The high similarity between the structures and sequences indicated that the available crystal structures of hCG and hFSH can be used as templates for fish GTH subunits α and β. Finally, superposing the overall sturgeon GTHs onto hCG and hFSH (data not shown) gave RMSD of 0.74 to 2.14 Å, leading to the conclusion that hFSH and hCG are very good templates for the conformation of stGTHs.

### Development and validation of ELISAs for stFSH and stLH

Competitive ELISAs were developed to determine the levels of stFSH and stLH in sturgeon pituitary and plasma samples, using stFSHβα and stLHβα as standards (Product C in Fig 1), stFSHβ or stLHβ (Product E in Fig 1) for coating, and specific primary antisera against stFSHβ or stLHβ for differential detection.

**Fig 4:**
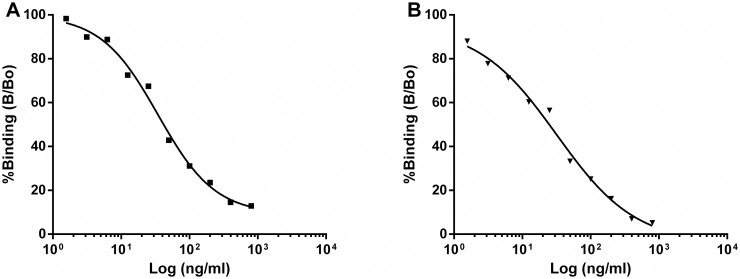
Competitive binding curves for Russian sturgeon FSH and LH standards. Typical standard curve for the measurement of Russian sturgeon FSH (A) and LH (B).

The standard curve ranged from 1.5625 to 800 ng/mL for both stFSH and stLH (Fig 4A and 4B, respectively. Under the described routine conditions, all standard curves typically showed a sigmoidal dose response (Fig 4). Detection threshold was defined as the amount of stLH or stFSH needed to reduce the optical density determined in their absence by two standard deviations. For measurement of stLH, the sensitivity of the assay was 218 pg/mL, with the optical density decreasing as a linear function of LH concentration (r^2^ = 0.90; Fig 4A). The sensitivity of the assay for stFSH was 1.56 ng/mL, with the optical density decreasing as a linear function of FSH concentration (r^2^ = 0.95; Fig 4A). The intra-assay CV, calculated by measuring replicates of the same sample in the assay, was estimated at 5.9% for stLH and 6.3% for stFSH. The inter-assay CV, calculated by measuring replicates of the same sample in different assays, was 17.7% and 9.2% for stFSH and stLH, respectively.

**Fig 5:**
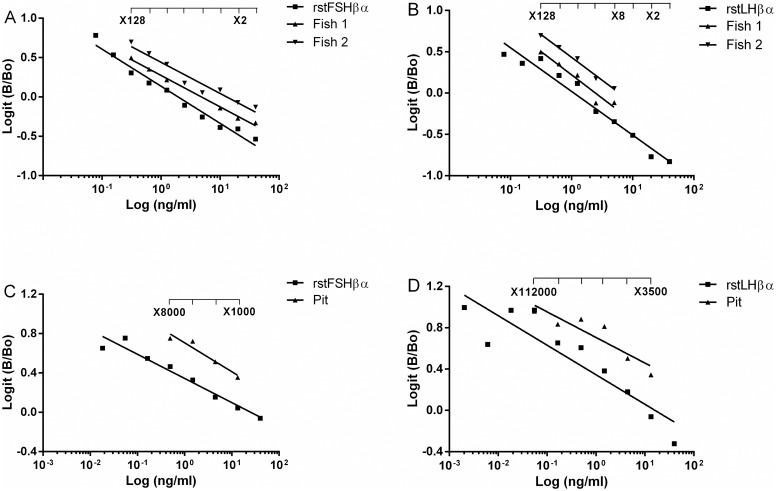
Parallelism between the standard curves of ELISA for the measurements of Russian sturgeon gonadotropins. Russian sturgeon FSH (A) or LH (B) and displacement curves obtained with serial dilutions of plasma from a male (Fish 1) and female (Fish 2). (C) Displacement curves obtained with serial dilutions of pituitary extract of Russian sturgeon FSH and (D) Pituitary extract of Russian sturgeon LH.

Validation of the assay for the determination of stFSH and stLH was performed by testing the parallelism between the standard curves and displacement curves obtained by serial dilutions of sturgeon plasma and pituitary. Serial dilutions of plasma from sturgeon were found to parallel both stLH and stFSH standard curves (Fig 5A and 5B). The slope of the displacement curve obtained with rstFSHβα (slope ± SEM, −0.5287 ± 0.086) was not significantly different from that obtained with native plasma FSH (slope ± SEM, −0.3943 ± 0.012). The slope of the displacement curve obtained with rstLHβα (slope ± SEM, −0.4712 ± 0.063) was not significantly different from that obtained with native plasma LH (slope ± SEM, −0.3954 ± 0.095; Fig 5A). Also for the pituitaries the slope of the displacement curve obtained with stFSHβα (slope ± SEM, -0.242 ± 0.030) was not significantly different from that obtained with native pituitary FSH (slope ± SEM, -0.293 ± 0.054). The slope of the displacement curve obtained with stLHβα (slope ± SEM, -0.249 ± 0.050) was not significantly different from that obtained with native pituitary LH (slope ± SEM, -0.247 ± 0.055). Sturgeon pituitaries were estimated to contain 420 ng LH/mg and 120 ng FSH/mg fresh pituitary tissue (or 63 mg LH/gland and 18 mg FSH/gland, at an average gland weight in 7-year-old females of 150 mg). These values are in the same order of magnitude as those reported for GTH content in the pituitary of the white sturgeon [29]. It should be noted, however, that these values correspond to females during a specific reproductive phase, and they are likely to show large oscillations at different seasons.

### Immunohistochemistry of the sturgeon pituitary

**Fig 6:**
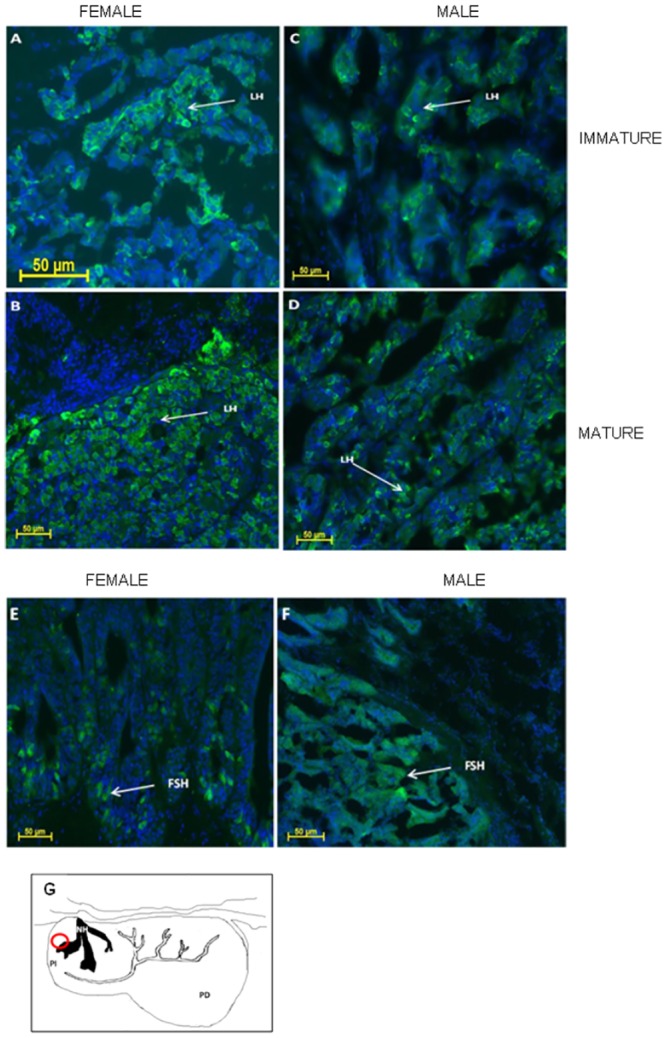
Immunofluorescence localization of stLHβ in sagittal sections of pituitary. Pituitaries from: immature female Russian Sturgeon at 4-year old (A; GSI = 3.2%), mature female at 9-year age (B; GSI = 9.2%), immature male at 2-year old (C; GSI = 0.7%); mature male at 5-year old (D; GSI = 1.2%); Immunofluorescence localization of stFSHβ in sagittal sections of pituitary from: mature female at 9-year age (E; GSI = 9.2%) and mature male at 9-year old (F; GSI = 1.2%). Schematic representation of sturgeon pituitary. The red circle mark the location of the imaged area within the pituitary (G): NH–neurohypophysis, PI–pars intermedia, PD–pars distalis.

To further validate the specific antibodies raised against the recombinant stFSHβ and stLHβ, and to identify the specific localization of each of the GTHs in the sturgeon pituitary, we used the novel specific antibodies for immunochemical staining. The pituitaries from a 9-year-old (mature) female (GSI = 9.2%), a 4-year-old (immature) female (GSI = 3.2%), a 5-year-old (mature) male (GSI = 1.2%) and a 1-year-old immature male (GSI = 0.7%) were generally processed according to [22]. Immunofluorescence localization revealed the existence of both GTHs (stFSHβ and stLHβ) in mature sturgeon pituitaries (both females and males; Fig 6). Significant differences in expression were revealed between immature and mature sturgeon pituitaries: LH cell numbers were higher in mature females and males (Fig 6B and 6D) than in immature females and males (Fig 6A and 6C). Mature female and male sturgeons exhibited FSH-positive cells in their pituitaries (Fig 6E and 6F).

### *In-vitro* biological activity of recombinant stLHβα and stLHβα

**Fig 7:**
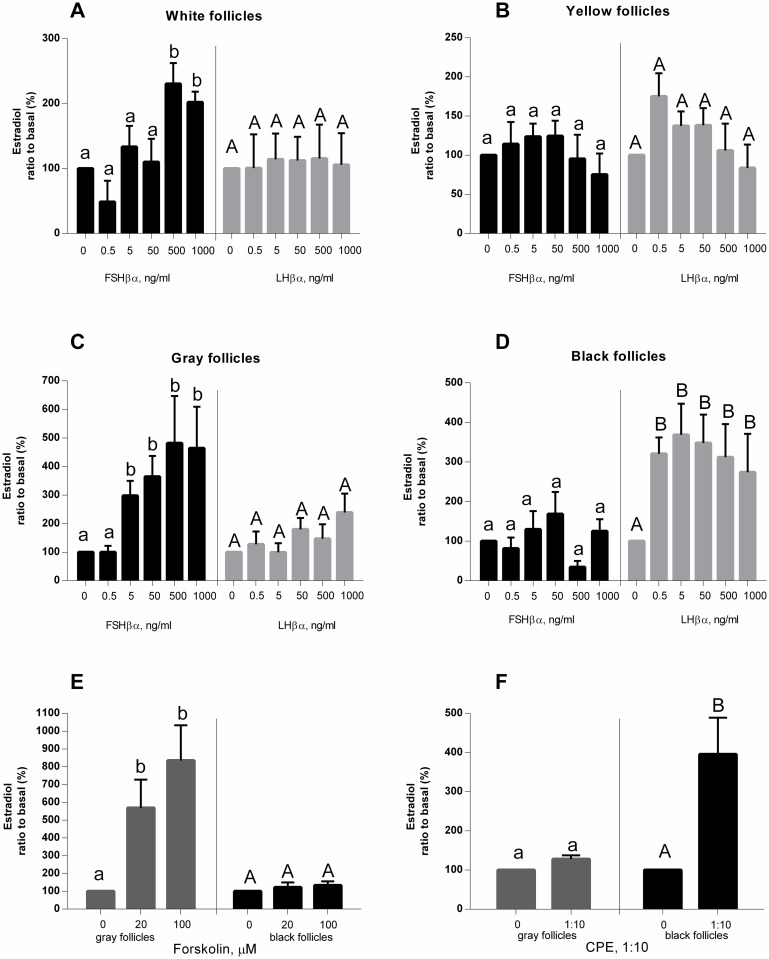
Secretion of estradiol from sturgeon ovaries. Sturgeon ovaries containing white (A), yellow (B), gray (C) or black (D) oocytes from a mature female at 10-year age, in response to various doses of recombinant sturgeon FSHβα, rsLHβα, adenylate cyclase activator (Forskolin, FSK;E) or carp pituitary extract (CPE; F).

The release of E2 from the ovaries of sturgeon was used to assay the role of recombinant stFSHβα and stLHβα. Table 1 describes the different stages of the ovarian follicles. Follicles from pre-vitellogenic ovary secreted E2 in response to stFSHβα, but not stLHβα (Fig 7A). Dose-dependent secretion of E2 was found when mid-vitellogenic follicles (gray oocytes) were exposed to stFSHβα, with a minimal effective dose of 5 ng/mL, and a maximal response at 500 ng/mL (Fig 7C). Mid-vitellogenic follicles also secreted E2 in response to high doses of stLHβα (Fig 7C). Only females carrying black follicles secreted E2 in response to both stFSHβα and stLHβα. The recombinant β subunits alone did not show any stimulation of E2 production (data not shown). Gray oocytes responded better to FSH stimulation, while black oocytes from a mature female responded more effectively to stimulation by LH (Fig 7C and 7D). Our results showed that the recombinant FSH and LH elicits different secretion levels of E2 in mature vs. immature females, suggesting a differential function for LH and FSH during ovarian development, where, as in the case of salmonids, LH is predominant in the mature female sturgeon. Mature (gray and black) follicles were exposed to graded doses of forskolin (an activator of the cAMP pathway) or carp pituitary extract (CPE). Forskolin increased E2 secretion from gray follicles, but not from black ones, while CPE, which contains more LH than FSH, increased E2 secretion only from black follicles (Fig 7E and 7F).

**Fig 8:**
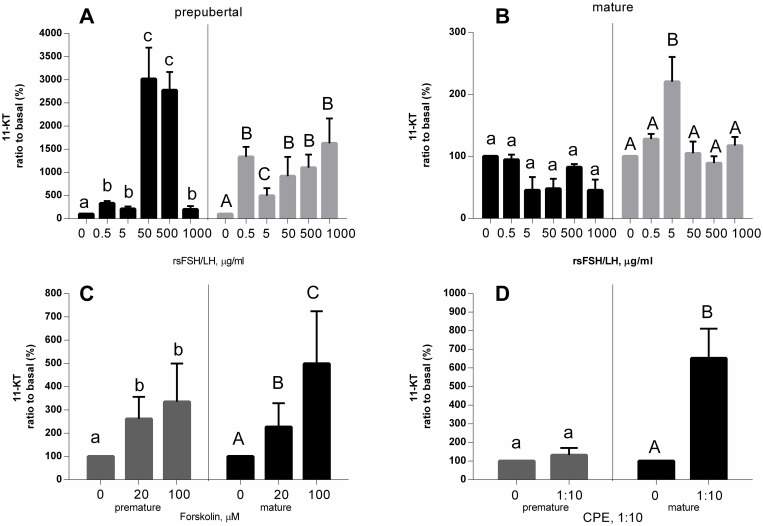
Secretion of 11-KT from sturgeon testes. Testes from pre-mature (A) or mature (B) sturgeon, in response to various doses of recombinant sturgeon FSHβα, rsLHβα, adenylate cyclase activator (Forskolin, FSK; C) or carp pituitary extract (CPE; D).

Interestingly, prepubertal testes were responsive to both recombinant stGTHs. However, mature testes were more sensitive to LH than FSH secretion (Fig 8A and 8B). Similarly, CPE, which contains high amounts of LH and low FSH, was more effective in the release of 11-KT from mature testes than from prepubertal ones. However, the adenylate cyclase activator forskolin enhanced the release of 11-KT in a similar fashion and to a similar degree from prepubertal and mature testes (Fig 8C and 8D).

### Effect of combined GnRH and testosterone treatment on plasma hormone levels and gonadal staging in mature female Russian sturgeon

**Fig 9:**
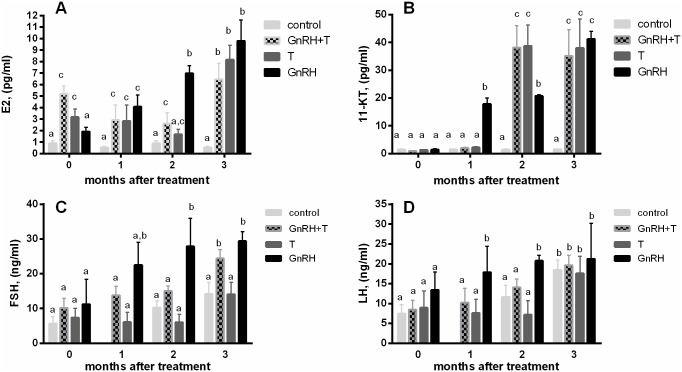
Effect of hormonal treatments on E2, 11-KT, FSH and LH plasma level in 6-year-old Russian sturgeon females. The control group was injected with 0.2 mL/kg saline. The first treatment group was injected (IM) with salmon GnRH analog ([D-Arg^6^,Pro^9^- NEt]-salmon GnRH; sGnRHa) in slow-release implants (Evac) up to a final dose of 10 μg/kg. Fish from the second group received testosterone (T) as food supplements (60 mg/kg food; 0.4% BW food per day). The third group received both GnRH implants and T as food supplements, at the above concentrations. Each bar represents the mean ± SEM. Different letters indicate that treatments have significantly different effects (ANOVA followed by Newman–Keuls test, (p<0.05).

These experiments were conducted from March to June, the natural reproduction period for sturgeon, starting as soon as the water temperature reached 20°C. Fish were bled four times: at the beginning of the experiment, and monthly thereafter. Female fish fed with T-containing diets showed high levels of 11-KT in their plasma. However, the combination of GnRH + T did not affect the levels of the androgenic steroid (Fig 9B). A similar response pattern was evident for E2 secretion (Fig 9A). However, GnRH + T significantly enhanced FSH levels, while no change was present in LH release (Fig 9C and 9D). All of the fish were observed internally by endoscopy to determine their gonadal stage (according to [30]. Based on the endoscopy results we calculated how many females have shown to carry follicles at a more advanced stages in their ovaries. In the control group, only 3 females out of 10 had more advanced stages after 10 months of duration of the experiment (Table 2). However, in the GnRH group, at the beginning of the experiment, 9 females were at the pre-vitellogenic stage and only one female carried white follicles, while 10 months later 5 females carried white follicles (4 more than 10 months before), 2 females carried yellow follicles and 2 females carried gray follicles (80%). In the group that was fed T, only 4 females out of 12 (3 females carried white follicles and one carried yellow follicles; 33%) showed any advanced changes. In fish that were injected with GnRH and administered T, 9 out of 13 females showed more advanced stages (7 females carried white follicles, one female carried yellow follicles and one gray follicles; 69%; Table 2). These results suggest that slow-release implants of GnRH may enhance development of the sturgeon ovary, while T has no significant effect at all.

**Table 2:** Effect of combined GnRH and testosterone treatment on gonadal staging.

Stage/Group	Control(n = 10)		GnRH(n = 10)		T(n = 12)		GnRH + T(n = 13)	
	Time0	10 months later	Time0	10 months later	Time0	10 months later	Time0	10 months later
**Pre-vitellogenic**	5	3	9	1	10	6	11	2
**White follicles**	5	4	1	5	2	5	2	9
**Yellow follicles**	0	2	0	2	0	1	0	1
**Gray follicles**	0	1	0	2	0	0	0	1

Six-year-old female sturgeon were injected with saline (control group) or GnRH implants (10 mg/kg; GnRH), were fed testosterone (60 mg/kg feed; T), or injected with GnRH implants and fed T at the indicated doses (GnRH + T). Ovaries were observed endoscopically at the beginning of the experiment (time 0) and 10 months later. The number of females at each of the specified ovarian stage (according to the stages defined in Table 1) is presented.

## Discussion

Our current understanding of the hormonal control of the hypothalamic–pituitary–gonadal axis in aquatic vertebrates stems almost entirely from work on advanced teleosts. In the current work, we studied several steps along the axis that leads to reproduction in the Russian sturgeon—an ancient species that belongs to the chondrostei group of bony fish.

In the present study, we demonstrated that (a) biologically active recombinant sturgeon GTHs can be produced in large scale by the yeast Pichia pastoris; (b) the hormones have the potency to stimulate the secretion of steroids *in vivo* and (c) GnRH treatment was more efficient in accelerating the readiness of the ovary than treatment by T alone or in combination with GnRH.

In our previous work [16], we cloned the sequences of the Russian sturgeon GTH subunits. These sequences were introduced into the methylotrophic yeast *P*. *pastoris* expression system to produce the respective recombinant stLHβα and stFSHβα proteins. This system produced a sufficient quantity of correctly folded recombinant proteins. Consequently, these proteins were used as antigenic agents for the production of specific antibodies as well as standard proteins, both essential for the development of hormone-specific ELISAs.

The stFSH and stLH glycoprotein hormones are dimers consisting of a common α subunit and a hormone-specific β subunit, aligned in opposite directions, head to tail.

Given the size of these fish, it would have been easy to extract and purify pituitary LH and FSH, but since sturgeon are becoming extinct, the production of recombinant LH and FSH is the most forward-looking approach. *P*. *pastoris* has been recently used, by us and others, as a heterologous expression system for the production of recombinant fish GTHs due to its efficient secretion, glycosylation potential, high expression level, and high cell density [2,31]. To overcome the bias of codon usage, we optimized our native gene sequence *in silico* to match the heterologous gene expression system *P*.*pastoris*. For this purpose, we used preferred codons, eliminated AT-rich stretches and adjusted the G + C content. The C-terminal region of the α subunit and the N-terminal region of the β subunit of the GTHs were shown to be crucial for receptor binding [32,33]. Therefore, the mature protein coding sequences were joined to form a fusion gene that encodes a hitched polypeptide consisting of the β chain forming the N-terminal part and the α chain forming the C-terminal part of the sturgeon recombinant GTHs. Single-chain GTHs have previously been produced in different teleosts, including Nile tilapia [14,15], Japanese eel (*Anguilla japonica*) [34], Manchurian trout (*Brachymystax lenok*) [35], zebrafish (*Danio rerio*) [36], sea bream (*Sparus aurata*) [37], catfish (*Ictalurus punctatus*) [38], Senegalese sole [39], seabass [40], sea lamprey (*Petromyzon marinus*) [41] and recently in the yellowtail kingfish (*Seriola lalandi*) (Sanchis-Benlloch et al., 2016). Adding a His^6^-tag to the protein, which does not show negative effects on the bioactivity of the hormones [14,15], allowed us to purify the recombinant GTHs from the culture medium. Western-blot analysis using novel sturgeon anti-FSHβ and anti-LHβ polyclonal antibodies, or anti-His antibody, following PNGase F treatment revealed that both recombinant stGTHs were extensively glycosylated by asparagine-linked glycans. These findings are consistent with the *in-silico* detection of two *N*-glycosylation sites in stFSHβ, one in stLHβ, and two in the stGPα subunits [16]. Deglycosylation of sturgeon recombinant and pituitary GTHs with PNGase F, which hydrolyzes all types of *N*-glycan chains, reduced each of the glycosylated forms to its nascent translated protein. This implies that the carbohydrate modifications on the mature proteins occur exclusively through *N*-linked (and not *O*-linked) glycosylation, similar to findings for other fish species, such as Japanese eel FSH [42], tilapia LH [14] and tilapia FSH [15]. In contrast, mammalian glycoprotein hormones carry both *O*-linked and *N*-linked oligosaccharides [43]. The sugar moiety localized on the α subunit of stGTHs has been shown to play an important role in both stabilizing GTH structure and function, and is required to elicit cellular response [44]. It seems that the sugar moieties of fish GTHs are different from those of mammals in terms of structure and role, but this issue requires further study.

The sturgeon α subunit consists of three β hairpins, two of which extend from one end of the molecule (L1 and L3), while the other (L2) forms a longer loop at the opposite end of the molecule. The L2 loop of the α subunit includes a helical segment (1.5-turn α helix) that runs nearly perpendicular to the β strands. At the N terminus of both β subunits, a 3-turn α helix is formed. The FSHβ and LHβ conformations consist of three β hairpins, two extending from one end of the molecule (L1 and L3), and the other (L2) at the opposite end. Α loop at the C terminus is observed which tends to wrap around the α subunit. This loop is referred to as the seatbelt region, which is a key part of the binding site of the FSHβ subunit (2).

The ELISA developed for Russian sturgeon in the current study to specifically measure FSH and LH levels was validated for both plasma and pituitary samples by testing for parallelism with the standard curve. The results indicated that curves of the native stFSH and stLH, in both the blood and the pituitary, indeed paralleled those of the recombinant FSH–LH heterodimers used in the standard curve, and therefore were immunologically similar.

It is important to note that to date, methods for measuring GTHs are currently available for only several teleost species. To expand the availability of quantitative analysis tools for basal teleosts, like sturgeons, is of great interest. We developed two new competitive ELISAs for the measurement of stLH and stFSH. There are only a few fish species to which RIA and ELISA methods for the measurement of GTHs can be compared in terms of precision and detection level. A RIA for stGTHs was developed in the 1990s [29]. The minimum detectable concentration for the stGTH I (= FSH) RIA was 0.84 ng/mL while that of stGTH II (= LH) RIA was 1.25 ng/mL [29]. The gonadotropin levels as measured by RIA were similar to those found under the current study using competitive ELISA. Although the sensitivities of ELISA and RIA procedures for the measurement of GTHs seem to be of the same order of magnitude, the ELISA is the first choice option due to its practical advantages over the RIA.

stFSH and stLH cells were located in the ventral and dorsal pars distalis of the pituitary, and some bordered the ventral area of the pars intermedia. Similar areas of gonadotropin secreting cells were also found in other Acipenser species like the Chinese sturgeon [45], *A*. *stellatus* [46] and *A*. *baeri* [47].

To determine the differential role of each of the GTHs in male and female sturgeon, we incubated gonadal fragments, at different stages, with graded doses of stFSH or stLH ranging from 0 to 1000 ng/mL. FSH was clearly more effective than LH in inducing E2 production and secretion from early-vitellogenic (white) or mid-vitellogenic (gray) than from mature (black) follicles; LH was very efficient at inducing E2 production in mature follicles. These findings suggest that, similar to the conventional model in fish, FSH has a significant role in the first stages of gametogenesis and vitellogenesis, whereas LH is more important at the final stages of oocyte maturation and ovulation [2]. This is consistent with previous results showing that immature sturgeon females exhibit seasonal changes in E2 secretion, with levels rising with water temperature in the spring as vitellogenesis advances [48,49]. FSH succeeded to stimulate E2 production in gray follicles as well. These results are in corroboration with the results in the white sturgeon where higher levels of FSH were found in pre-vitellogenic and vitellogenic follicles, while higher levels of LH were found in ovulation, although FSH was also found at the end of maturation [29]. Our immunohistochemistry analysis also showed FSH cells in mature females. High levels of FSH during the final stages of follicle maturation have also been observed in other fish species for instance tilapia [7], trout [50], carp [51] and salmonids [52]. Although no direct role was found for FSH at these stages, it may be assumed that these high levels are related to the recruitment of a new generation of follicles into early vitellogenesis, or to oogenesis that occurs before the rise of a new reproductive cycle. These processes are common to fish baring either synchronous ovaries (like sturgeon) or asynchronous ovaries.

FSH increased 11-KT levels in prepubertal male gonads at low doses, whereas LH was effective only at higher concentrations. However, LH was the only GTH to efficiently increase 11-KT production in mature sturgeon testes. These results corroborate the notion that in sturgeon, like in other fish species, FSH plays a major regulatory role during early stages of spermatogenesis, whereas LH is mainly involved in the final stages of maturation. In fish, it is accepted that both pituitary GTHs stimulate gonadal sex steroid hormone production directly by activating Leydig cells (reviewed by [53].

In farmed Russian sturgeon, most males mature at the age of 4 years, and females reach first sexual maturity between 6 and 12 years [16,48]. This is similar to the white sturgeon (*Acipenser transmontanus*), where farmed males can reach puberty at the age of 3–4 years, while females will reach puberty at the age of 6–14 years [12]. In the white sturgeon, pituitary and plasma concentrations of FSH were shown to be higher than those of LH during vitellogenesis and early stages of spermatogenesis. Conversely, pituitary and plasma concentrations of LH were advanced than those of FSH during ovulation and spermiation [29]. In the Russian sturgeon, the expression of FSHβ in immature (2 to 4 years of age) females and males increased with age, while LHβ levels were constant [16]. The expression of Chinese sturgeon (*A*. *sinensis*) FSHβ was not detected at all in immature males or females [45].

It has been well documented, in mammals and fish, that both FSH and LH signal their transduction through the cAMP/PKA (protein kinase A) pathway [2]. Hence, it was not surprising to see an increase in E2 and 11-KT levels in females and males, respectively, after exposure of gonads to the PKA activator forskolin. It is also well established that in sturgeon species, ovulation and spermiation can be induced by CPE. To induce spermiation, sturgeon were given a single injection of 1.5 mg CPE/kg BW, and semen was collected 30 h later (12). In the sterlet (*A*. *ruthenus*), spermiation rate was 100% in fish injected with CPE [54]. Captive white sturgeon females were induced to ovulate by administration of CPE when they reached a responsive stage [12]. Indeed, CPE increased 11-KT secretion from mature, but not from prepubertal sturgeon testes, and from ovaries containing black—but not gray—follicles.

In the present study, GnRH alone or GnRH + T were able to induce FSH release, while GnRH alone and GnRH + T were both sufficient to advance the development of mature ovaries. These results are in line with other studies in juvenile or sexually resting teleosts that showed that GnRHa more effectively stimulates pituitary LH/LHβ when combined with T [55–58]. In the pink salmon (*Oncorhynchus gorbuscha*), neither GnRH nor T resulted in significantly greater ovarian or testicular growth, but their co-administration significantly increased ovarian growth after 5 months [59]. The combination of GnRH + T also increased E2 levels in the Russian sturgeon. The mechanisms underlying this androgen-induced increase in the pituitary response to GnRH may involve a positive effect of E2 on GnRH receptor gene expression [60], upregulation of GnRH receptor by GnRH [61] and/or a modulation of GnRH receptor signaling pathways [62]. Overall, our findings reinforce the notion that T plays an important role in the activation of FSH signaling during the initiation of puberty in teleosts and thus, it must be considered in treatments designed to stimulate the reproductive axis of prepubertal sturgeon. A similar situation was recently found in the sablefish (*Anoplopoma fimbria*) [63].

In the current study, treatment with a combination of T and GnRH did not result in significant stimulation of LH in juvenile females, which was surprising because in other immature fishes, pituitary LH content is successfully elevated by GnRH + T steroid treatments [64–66].

11-KT is an oxidized form of T that contains a keto group at position 11. 11-KT, usually considered as male-specific androgen in teleost fish, has also been found in several female fishes [67]. Moreover, although the predominant androgen in most sexually recrudescent females is T, in sturgeon, 11-oxygenated androgens are present at levels as high as, or higher than, those of T [68]. Our results corroborate this since we found high levels of 11-KT in females, especially in those injected with T, showing that the enzyme that converts T to 11-KT—11β-hydroxylase is present in female sturgeon.

Pituitary concentrations of both FSH and LH were significantly higher in juvenile or vitellogenic white sturgeon females that were treated with T implants. However, juvenile sturgeon which were injected intraperitoneally with GnRHa showed no change in plasma concentrations of neither of the GTHs [69]. These variations are probably due to morphological or endocrine differences between the white and the Russian sturgeon resulting in different hormone levels at specific reproductive stages; yet these assumptions need further confirmation.

In summary, we described the establishment of a biologically potent recombinant sturgeon FSH and LH, the validation of a homologous ELISA based on recombinant gonadotropins and specific antibodies. In addition, we describe a series of studies aimed at improving our understanding of the regulation of the reproductive axis in prepubertal female sturgeon, which would ultimately lead to the development of methods to control the age of puberty.
